# 新型EGLN1基因突变致遗传性红细胞增多症1例

**DOI:** 10.3760/cma.j.cn121090-20251114-00531

**Published:** 2026-03

**Authors:** 启国 张, 文静 傅, 春 凌, 文强 鲍, 长志 赵, 其川 金

**Affiliations:** 安徽医科大学附属滁州医院，滁州市第一人民医院，滁州 239001 The First People's Hospital of Chuzhou, Chuzhou Hospital Affiliated to Anhui Medical University, Chuzhou 239001, China

患者，男，35岁；红细胞持续增多2年，HGB波动在191～201 g/L，偶有头昏，有时耳鸣。门诊血常规：WBC 6.93×10^9^/L，HGB 203 g/L，RBC 6.45×10^12^/L，血细胞比容（Hct）54％，PLT 279×10^9^/L。血清促红细胞生成素（sEPO）15.3 U/L（参考值4.3～29.0 U/L）。JAK2 V617F及JAK2 EXON12突变阴性。血清铁、铁蛋白正常。体检示肥胖［体重指数（BMI）31.1 kg/m^2^］，呈轻度多血质貌。B超：脂肪肝、胆囊壁毛糙，脾脏大小正常；心脏超声正常。外周血遗传学全外显子测序示：EGLN1基因chr1：231557220 NM_022051.3，Exon 1/5，c.415G>A（p.Ala139Thr）杂合突变。家系Sanger测序验证该变异遗传自父亲。先证者口腔黏膜拭子检测到该位点变异，变异等位基因频率（VAF）为39.9％。EGLN1蛋白三维结构分析显示，p.Ala139Thr周围的氨基酸残基及氢键数量未见变化，丙氨酸（Ala）是非极性疏水性氨基酸，苏氨酸（Thr）为极性中性（亲水性）氨基酸，导致该位点局部亲水性能发生改变（图1）。家族史：先证者母亲2019年因脑梗死及高血压去世，生前血常规正常；父亲平时无不适，此次家系调查血常规示：WBC 6.98×10^9^/L，HGB 177 g/L，RBC 5.67×10^12^/L，Hct 51％，PLT 279×10^9^/L。姐姐及其丈夫育一女，均体健。个人史及既往史：职业为厨师，有重度吸烟史；有高血压、糖尿病（口服达格列净2年）、高脂血症、高尿酸血症及胰腺炎病史；睡眠时有打鼾。骨髓象：增生活跃，粒红比为1.43∶1；中、晚幼红细胞比例分别为10.5％、23.5％，中晚幼粒细胞比例稍偏高；全片见巨核细胞59个，分类25个，其中颗粒巨核细胞19个，产板巨核细胞5个，裸核巨核细胞1个。骨髓活检除网状纤维化（MF）1级，未见骨髓增殖性肿瘤（MPN）典型病理改变。染色体核型：46, XY[20]。最终诊断：遗传性红细胞增多症（ECYT 3型）。

**图1:**
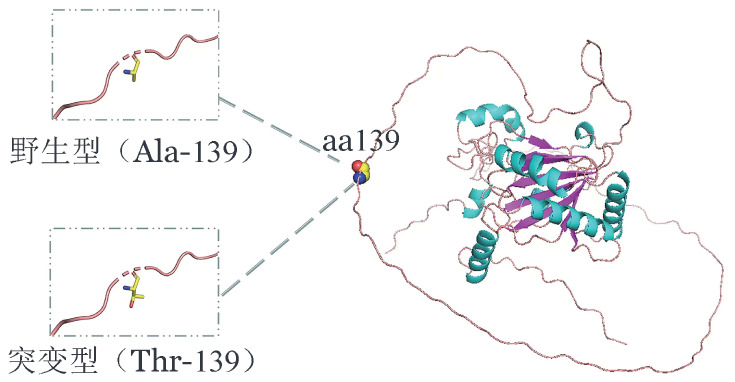
EGLN1蛋白质三维结构示意图，右侧蓝色示α螺旋，紫色示β折叠，粉色示Loop结构。139位氨基酸（aa 139）所在位置以空间填充模型标出。左侧虚线框黄色示碳（C）原子，蓝色示氮（N）原子，红色示氧（O）原子。p.Ala139Thr位点周围的氨基酸残基空间排布及氢键（红色虚线）数量未见明显变化

给予短期口服羟基脲进行降细胞治疗，辅助口服银杏叶提取物及甲钴胺，头昏、耳鸣症状稍改善。嘱其进行生活方式调整（包括戒烟、减重），并口服阿司匹林预防血栓。目前患者无明显不适，末次随访HGB 185 g/L、Hct 55％。

讨论：JAK2基因未突变的红细胞增多症涵盖了一系列异质性的遗传与获得性病症。诊断时需考虑罕见遗传性病因，全外显子测序是明确诊断的关键。自2006年Percy等首次报道EGLN1突变与红细胞增多症关联以来，国内文献鲜见相关报道。本例EGLN1基因c.415G>A（p.Ala139Thr）突变在gnomAD等公共人群数据库中未见收录，丰富了ECYT3型的致病突变谱。ECYT3型通常呈良性病程，治疗应以缓解症状和预防血栓为主，注意个体化策略，避免过度细胞减灭治疗。

